# Underrepresentation of Women in Recent Landmark Kidney Trials: The Gender Gap Prevails

**DOI:** 10.1016/j.ekir.2022.08.022

**Published:** 2022-09-02

**Authors:** Amanda J. Vinson, David Collister, Sofia Ahmed, Karthik Tennankore

**Affiliations:** 1 Division of Nephrology, Department of Medicine, Dalhousie University, Nova Scotia, Canada; 2 Nova Scotia Health, Halifax, Canada; 3 University of Alberta Faculty of Medicine and Dentistry, Medicine, Alberta, Canada; 4 Cumming School of Medicine, University of Calgary, Calgary, Alberta, Canada; 5 Libin Cardiovascular Institute, Calgary, Alberta, Canada; 6 O'Brien Institute of Public Health, Calgary, Alberta, Canada; 7 Alberta Kidney Disease Network, Calgary, Alberta, Canada; 8 Population Health Research Institute, Ontario, Canada

**Keywords:** cardiovascular, disparity, gender, kidney, participation, sex, trials

## Introduction

With the recent development of sodium glucose cotransporter-2 inhibitors (SGLT-2i), glucagon-like peptide-1 receptor agonists (GLP1-RA), and nonsteroidal mineralocorticoid receptor antagonists (MRA), there has been a surge of high-quality randomized controlled trials demonstrating substantial benefits of these agents for persons with kidney disease, after a period of relative stagnation.^1^ These novel agents significantly reduce the risk of cardiovascular events and/or chronic kidney disease progression; benefits have been demonstrated in patients with diabetic kidney disease; and specific to SGLT-2i, patients with nondiabetic kidney disease.

Despite the benefits, earlier studies have raised concerns about potential sex-disparate effects of SGLT-2i in women and men.^2^ A 2016 meta-analysis of 26 SGLT-2i trials demonstrated a relatively smaller reduction in cardiovascular mortality when the proportion of women enrolled in the trials increased.^3^ In addition to sex-based differences in efficacy, women experience a higher proportion of some drug-related adverse events with SGLT2i (primarily genital and urinary infections) compared with men.^4^^,^^5^ Sex-specific differences in efficacy have also been reported with GLP1-RAs, namely, a larger relative reduction in cardiovascular events in women compared with men,^6^ but more frequent adverse events (particularly gastrointestinal events). Finally, whereas potential sex differences in the effects of finerenone (a nonsteroidal MRA) have yet to be examined, spironolactone (a steroidal MRA) has been associated with significant disparate effects on all-cause mortality among men and women with heart failure and preserved ejection fraction.^7^

The study of sex-specific treatment efficacy and safety has historically been challenging due to the prevailing underrepresentation of women in clinical trials. This has led to gendered strategies to promote recruitment of women^8^ and national policies to increase the representation of women in research studies.^9^ It is currently recommended that an appropriate sample size of women be included in all phases of clinical trials to allow adequate effects of drug treatments to be evaluated.S1 Nevertheless, a review of cardiovascular trials from 2010 to 2017 demonstrated that only 38.2% of participants were women, and relative to their prevalence in the disease population, participation to prevalence ratio (PPR) (defined as the percentage of women in a trial divided by the percentage of women with a disease state in the general population) was 0.66 for studies of acute coronary syndrome, and only 0.48 for studies of heart failure; a PPR of 0.8 to 1.2 suggests comparable prevalence and good representation.S2^,^S3 A similar review in nephrology has not been performed, however it has recently been shown that the overall proportion of female enrollees in non sex-specific nephrology trials may be among the lowest of all specialties.S4

Therefore, we aimed to examine the PPR for women versus men included in major contemporary trials published in the leading medical journal, the *New England Journal of Medicine*, examining SGLT-2i, GLP1-RAs and nonsteroidal MRAs.

## Results

We included 8 trials examining SGLT-2i (*n* = 66,309; 22,792 women [36.0%]), 3 trials examining GLP1-RAs (*n* = 16,713; 5976 women [35.8%]), and 2 trials examining finerenone (*n* = 13,026; 3938 women [30.2%]). A summary of trial populations is presented in Supplementary Table S1.

**Figure 1:**
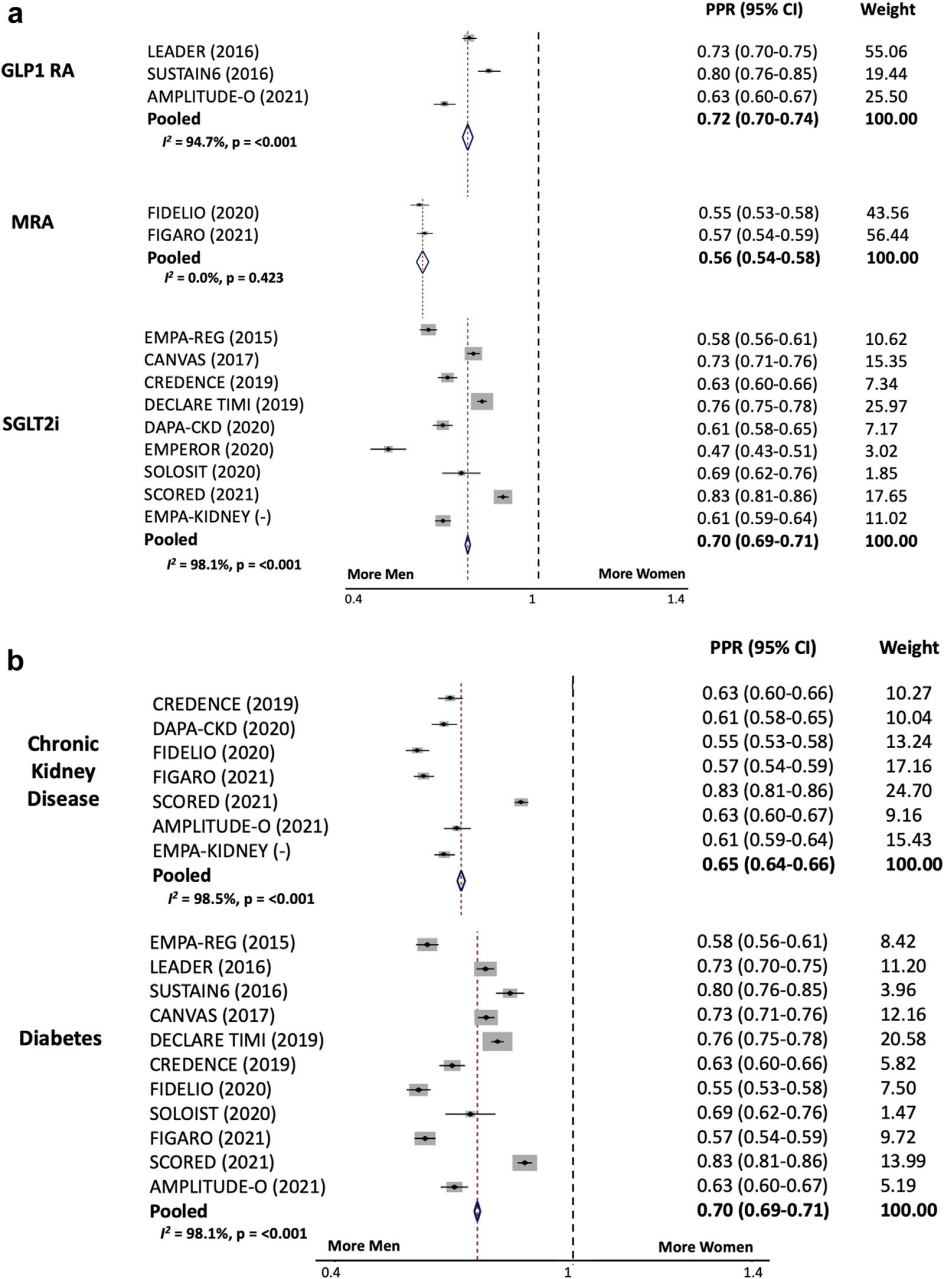
(a) Pooled Participation to Prevalence Ratio for Women versus Men in Landmark Trials of GLP1-RAs, MRAs and SGLT-2i from 2015 to 2021; (b) pooled participation to prevalence ratio for women versus men in landmark trials of GLP1-RAs, MRAs and SGLT-2i from 2015 to 2021, stratified by chronic kidney disease or diabetes as part of inclusion criteria. CI, confidence interval; GLP1-RA, glucagon like peptide 1 receptor agonists; MRA, mineralocorticoid receptor antagonists; SGLT-2i, sodium glucose cotransporter-2 inhibitors.

The proportion of women included in each trial ranged from 24.4% to 44.9%. The individual and pooled PPR for each study within each drug class is shown in Figure 1a.

The pooled PPR for SGLT-2i trials was 0.70 (95% confidence interval [CI] 0.69 to 0.71, for GLP1-RAs was 0.72 (95% CI 0.70 to 0.74), and for finerenone was 0.56 (95% CI 0.54 to 0.58). There was significant heterogeneity in the PPR for SGLT-2i trials (*I*^*2*^ 98.1%, *P*-value < 0.001) and for the GLP1 RA trials (*I*^*2*^ 94.7%, *P*-value < 0.001), but not for finerenone (*I*^*2*^ 0.0%, *P*-value 0.423), which may reflect including only 2 finerenone trials. Similar results were shown in a sensitivity analysis that did not correct for gender disparate disease prevalence, assuming equal prevalence in men and women (Supplementary Figure S1).

When examining the PPR for women in the above trials stratified by inclusion criteria, the pooled PPR for chronic kidney disease was 0.65 (95% CI 0.64 to 0.66), and for type II diabetes mellitus was 0.70, 95% CI 0.69 to 0.71 (with significant heterogeneity for both), Figure 1b.

Sex stratified results were presented in 3 out of 8 SGLT-2i trials, 1 out of 3 GLP1-RA trials, and 2 out of 2 trials of finerenone.

## Discussion

Herein, we show that in contemporary trials examining SGLT-2i, GLP1-RAs, and the nonsteroidal MRA, finerenone, women remain significantly underrepresented compared with male trial participants. A PPR of 0.8 to 1.2 suggests comparable prevalence and good representation;S2^,^S3 and only 2 of the 14 trials we examined had a PPR in this range. The *New England Journal of Medicine* was chosen for our review, because it has published the overwhelming majority of landmark trials in this field, with a wide reaching audience and impact factor greater than 90. The issue of biased recruitment might be even worse in journals with less rigorous publication criteria, which is an area for future study.

Though it remains unclear whether sex and/or gender influences the efficacy or safety of any of the above drug classes, general sex differences in pharmacokinetics, pharmacodynamics, body mass and composition, and drug bioavailability are well established.S5^,^S6 Earlier studies have shown sex-based differences in efficacy and safety profiles of various medications. For example, compared with men, women require lower dosages of antipsychotic drugs to control symptoms, have increased response to beta-blockers, increased mortality when digoxin is used to treat heart failure, and receive greater benefit with sacubitril/valsartan versus valsartan alone for heart failure with preserved ejection fraction.S7–S9 Whether differences in reported adverse events reflect true sex-specific differences in physiologic response to drug or alternatively, differential reporting patterns, remains to be seen.S10^,^S11 Although sex-based differences in the efficacy and adverse events with SGLT-2i, GLP1-RAs, and MRAs have been proposed, the underrepresentation of women in individual trials has made sex-based comparisons challenging. Possible sex differences in drug and outcome-specific efficacy, as well as side effect profiles underscore the need to either adequately power studies with both sexes to determine potential sex-by-treatment interactions, or at a minimum, to report sex-stratified data and sex-stratified outcome analyses to facilitate subsequent meta-analysis of sex differences in treatment response (as outlined in the Sex and Gender Equity in Research guidelines).S12

**Figure 2:**
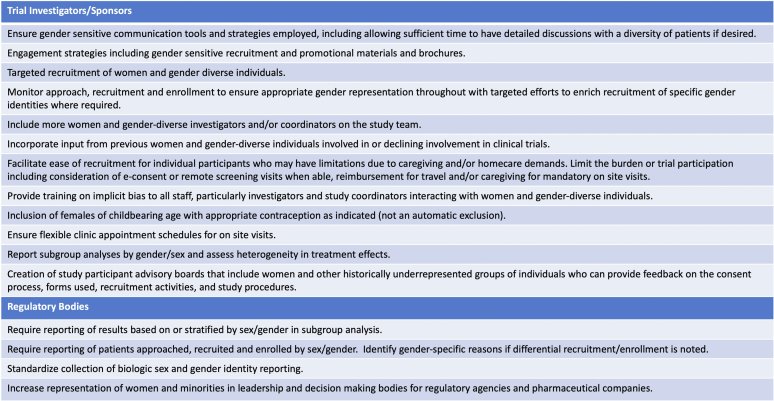
Potential strategies at the level of the investigator or regulatory bodies to bolster recruitment of women in clinical trials. Adapted from Bierer BE, et al. Cell Reports Medicine 3, 100553, April 19, 2022.

To enroll in a clinical trial, patients must be aware of the opportunity to participate and/or be approached, they must have access to centers participating in clinical trials, and they must understand and be comfortable with the clinical trial process.S2 Each of these may be influenced by patient or provider biases and the nature of their relationships, or by investigator or research coordinator’s communication approaches (verbal or written).S2 Women may perceive more harm from trial participation, are generally more risk adverse under stress, and have been shown to be more reluctant than men to participate in clinical trials.S13^,^S14 Furthermore, women of childbearing age may be excluded from study design due to the potential for pregnancy and/or breastfeeding. Barriers to women enrolling in cardiovascular disease trials are currently being explored in an ongoing qualitative and quantitative study, the WIN-Her Initiative Women Opt-In for Heart Research. This and similar studies may help identify gender-sensitive recruitment tools including patient and investigator gendered engagement strategies and communication tools. Future trials may need to monitor sex distribution throughout recruitment to ensure adequate representation across sexes, including specifically targeting recruitment of women or other genders as required to obtain balance and representation.S2 Strategies to bolster the recruitment of women in clinical trials are shown in Figure 2.

To achieve health equity for men and women and facilitate the identification of potentially important sex-differences in the efficacy and safety of medications, representative numbers of women must be prospectively included in all stages of clinical trials. Though the research community is commended for the bounty of recent evidence to reduce cardiovascular and kidney events in those at risk, women remain woefully underrepresented in recent landmark trials.

## Disclosure

All the authors declared no competing interests.

## Supplementary Material

**Supplemental Methods**

**Supplemental References**

**Figure S1.** Pooled participation to prevalence ratio for women versus men in landmark trials of GLP1-RAs, MRAs and SGLT-2i from 2015-2021, assuming equal disease prevalence in women and men

**Table S1.** Trial population, additional details

**Table S2.** Disease prevalence rates and inclusion rates by gender
