# Supplementary material for: Underrepresentation of Women in Recent Landmark Kidney Trials: The Gender Gap Prevails
Source: Kidney Int Rep. 2022 Sep 2;7(11):2526–9. doi: 10.1016/j.ekir.2022.08.022 (PMC9751686; doi:10.1016/j.ekir.2022.08.022)
Supplement: Supplementary File (PDF) [file mmc1.pdf]

Supplement

Supplemental Methods:

We included all trials published in the NEJM from 2015-2021 examining SGLT-2i, GLP1-RA or finerenone. We calculated the female to male sex ratio for each trial. The terms sex and gender are not synonymous, yet the terms are often used interchangeably in the literature, therefore we assumed women to mean female sex and men to mean male sex for the purposes of this study. We compared this to the female to male sex ratio for the prevalence of the dominant disease condition for the trial's inclusion criteria in the general population. If inclusion criteria comprised multiple conditions (e.g., CKD and DM2) the disease condition with the highest female prevalence in the general population was used to anti-conservatively calculate the PPR.

We calculated the prevalence-adjusted estimates for participation of women in trials by dividing the percentage of women among trial participants by the percentage of women in the disease population (PPR).[12] A PPR of near 1 indicates that the gender composition of the trial approximates the gender prevalence in the general population with that disease state. The disease prevalence rates used are shown in **Supplemental Table 2.** Global estimates were utilized for DM2 and CKD, and estimates from America were used for HF.

A weighted PPR for the included trials representing each drug class was determined using meta-regression. We also separately examined the PPR for studies with either CKD, DM2, of HF as an inclusion criteria. Heterogeneity in PPR by drug class and by inclusion criteria was evaluated by Higgins I^2 and the chi-square test of heterogeneity.

We repeated our analysis assuming a population prevalence for women of 50% for all inclusion criteria conditions (no difference in disease prevalence by gender).

Finally, the proportion of trials examining each drug class that included a sex-stratified outcome analysis was also reported.

Supplemental Table 1: Trial population, additional details

Trial	Drug	Regions Represented	Race	Age	Inclusion Criteria	Exclusion of Women of Child Bearing Potential (WOCBP)**	Primary Outcome
**EMPA-REG**	Empagliflozin	N. America (1394)<br>L. America (1081)<br>Europe (2885)<br>Asia (1347)<br>Africa (313)	White (5081)<br>Asian (1517)<br>Black (357)	63.2; 63.1	DM2 and high CV risk	Included if agree to contraceptive guidance and to submit to periodic pregnancy testing during the trial	Cardiovascular deaths, nonfatal myocardial infarction, or nonfatal stroke
**CANVAS**	Canagliflozin	N. America*<br>C. & S. America<br>Europe<br>Other	White (7944)<br>Black (336)<br>Asian (1284)<br>Other (578)	63.3	DM2 and high CV risk	Included if not agree to contraceptive guidance.	Cardiovascular deaths, nonfatal myocardial infarction, or nonfatal stroke
**CREDENCE**	Canagliflozin	N. America (1182)<br>C. & S. America (941)<br>Europe (864)<br>Other (1414)	White (2931)<br>Black (224)<br>Asian (877)<br>Other (369)	63.0	DM2 and CKD; eGFR 30 to <90 and uACR >300-5000; All on max tolerated RAASi	Included if agree to contraceptive guidance.	ESKD (dialysis, transplantation, or a sustained estimated eGFR of <15), doubling of serum creatinine level, or death from kidney or cardiovascular causes
**DECLARE-TIMI**	Dapagliflozin	N. America (5468)<br>Europe (7629)<br>L. America (1877)<br>Asia-Pacific (2186)	White (13653)<br>Black (603)<br>Asian (2303)<br>Other (601)	63.9; 64.0	DM2; CV risk	Included if agree to contraceptive guidance.	MACE and a composite of cardiovascular death or hospitalization for HF
**DAPA-CKD**	Dapagliflozin	Asia (1346)<br>Europe (1233)<br>N. America (813)<br>L. America (912)	White (2290)<br>Black (191)<br>Asian (1467)<br>Other (356)	61.8	eGFR 25-75 and uACR 200-5000; All on max tolerated RAASi	Included if agree to contraceptive guidance.	Sustained decline in the eGFR of at least 50%, ESKD, or death from kidney or cardiovascular causes
**EMPEROR**	Empagliflozin	N. America (425)<br>L. America (1286)<br>Europe (1353)<br>Asia (493)<br>Other (173)	White (2629)<br>Black (257)<br>Asian (672)<br>Other (172)	67.2; 66.5	HF class II, III, IV and EF ≤40%; All on max tolerated RAASi	Included if agree to contraceptive guidance.	Cardiovascular death or hospitalization for worsening HF
**SOLOIST**	Sotagliflozin	E. Europe (490)<br>W. Europe (310)<br>L. America (266)<br>N. America (80)<br>Other (76)	White (1139)<br>Black (50)<br>Asian (15)<br>Other (6)	69; 70	DM and recently hospitalized for worsening HF	Included if agree to contraceptive guidance and to submit to periodic pregnancy testing during the trial.	Cardiovascular deaths, hospitalizations for HF, and urgent visits for HF
**SCORED**	Sotagliflozin	E. Europe (3226)<br>W Europe (1420)<br>L. America (3172)<br>N. America (1491)<br>Other (1273)	White (8749)<br>Black (364)<br>Asian (682)<br>Other (666)	69	DM2 and CKD (eGFR 25-60) and CV risks	Included if agree to contraceptive guidance and to submit to periodic pregnancy testing during the trial.	Cardiovascular deaths, hospitalizations for HF, and urgent visits for HF Changed during the trial.
**EMPA-KIDNEY**	Empagliflozin	Europe (2648)<br>N. America (1717)<br>China/Malaysia (1632)<br>Japan (612)	White (3859)<br>Black (262)<br>Asian (2393)<br>Other (95)	63.8	eGFR ≥20 to <45 or ≥45 to < 90 and uACR ≥200; All on max tolerated RAASi	Included if agree to contraceptive guidance.	Cardiovascular or renal death, maintenance dialysis or transplant, sustained eGFR <10 or sustained ≥40% eGFR drop
**LEADER**	Liraglutide	Europe (3296)<br>N. America (2847)<br>Asia (711)<br>Other (2486)	White (7238)<br>Black (777)<br>Asian (936)<br>Other (389)	64.2; 64.4	DM2 and high CV risk;<br>Age ≥50 + established CKD or CV disease or age ≥60 and risk factors for CV disease	Included if agree to contraceptive guidance.	Cardiovascular death, nonfatal MI, or nonfatal stroke (non-inferiority)
**SUSTAIN6**	Semaglutide	Europe*<br>USA<br>Other	White (2736)<br>Black (221)<br>Asian (273)<br>Other (67)	64.6	DM2	Included if agree to contraceptive guidance.	Cardiovascular death, nonfatal myocardial infarction, or nonfatal stroke (non-inferiority)
**AMPLITUDE-O**	Efpeglenatide	Canada/US (1079)<br>Mexico/Central & S. America (924)<br>Europe (1285)	White (3534)	64.5	DM2 and either CV disease or CKD (eGFR 25-60) + CV risk	Included if agree to contraceptive guidance.	The first major adverse cardiovascular event (MACE; a composite of nonfatal myocardial infarction, nonfatal

		Other (788)					stroke, or death from cardiovascular or undetermined causes).
**FIDELIO**	Finerenone	N. America (944)<br>L. America (593)<br>Europe (2358)<br>Asia (1579)<br>Other (200)	White (3592)<br>Black (264)<br>Asian (1440)<br>Other (378)	65.6	CKD and DM2; uACR 30-300, eGFR 25-60, diabetic retinopathy, or uACR 300-5000, eGFR 25-75; All on max tolerated RAASi	Included if agree to contraceptive guidance. (≥2 effective methods of birth control, of which ≥1 is a physical barrier)	Kidney failure, a sustained decrease of at least 40% in eGFR from baseline, or death from renal causes
**FIGARO**	Finerenone	Europe (3504)<br>N. America (1107)<br>L. America (841)<br>Asia (1625)<br>Other (275)	White (5277)<br>Black (258)<br>Asian (1454)<br>Other (347)	64.1	CKD and DM2; uACR 30-300 and eGFR 25-90 or uACR 300-5000 and eGFR ≥60; All on max tolerated RAASi	Included if agree to contraceptive guidance. (≥2 effective methods of birth control, of which ≥1 is a physical barrier)	Cardiovascular death, nonfatal myocardial infarction, nonfatal stroke, hospitalization for HF

CKD (chronic kidney disease), DM2 (diabetes mellitus type 2), uACR (urinary albumin-creatinine ratio), RAASi (renin-angiotensin-aldosterone system inhibition), eGFR (estimated glomerular filtration rate), MACE (major adverse cardiac event), CV (cardiovascular), HF (heart failure), EF (ejection fraction)

*Breakdown by region not available.
**Pregnancy and breastfeeding were contraindications in all trials.

Supplemental Table 2: Disease prevalence rates and inclusion rates by gender

Trial	Drug Class	Women	Men	Total	Inclusion Criteria	Population Prevalence (%)	PPR	PPR if Population Prevalence 50%
**EMPA-REG**	SGLT-2i	2004	5016	7020	DM2	49 [24,25]	0.58	0.57
**CANVAS**		3633	6509	10142	DM2	49	0.73	0.72
**CREDENCE**		1494	2907	4401	CKD, DM2	54 [26 27]	0.63	0.68
**DECLARE-TIMI**		6422	10738	17160	DM2	49	0.76	0.75
**DAPA-CKD**		1425	2875	4304	CKD	54	0.61	0.66
**EMPEROR**		456	1411	1867	CHF	52.4 [11]	0.47	0.49
**SOLOIST**		412	810	1222	DM2	49	0.69	0.67
**SCORED**		4754	5830	10584	CKD, DM2	54	0.83	0.90
**EMPA-KIDNEY**		2192	4417	6609	CKD	54	0.61	0.66
**LEADER**	GLP1-RA	3337	6003	9340	DM2 + CV risk	49	0.73	0.71
**SUSTAIN6**		1295	2002	3297	DM2	49	0.80	0.79
**AMPLITUDE-O**		1344	2732	4076	DM2 ± CV disease or CKD + CV risk	49	0.63	0.63
**FIDELIO**	MRA	1691	3983	5674	CKD, DM2	54	0.55	0.60
**FIGARO**		2247	5105	7352	CKD, DM2	54	0.57	0.61

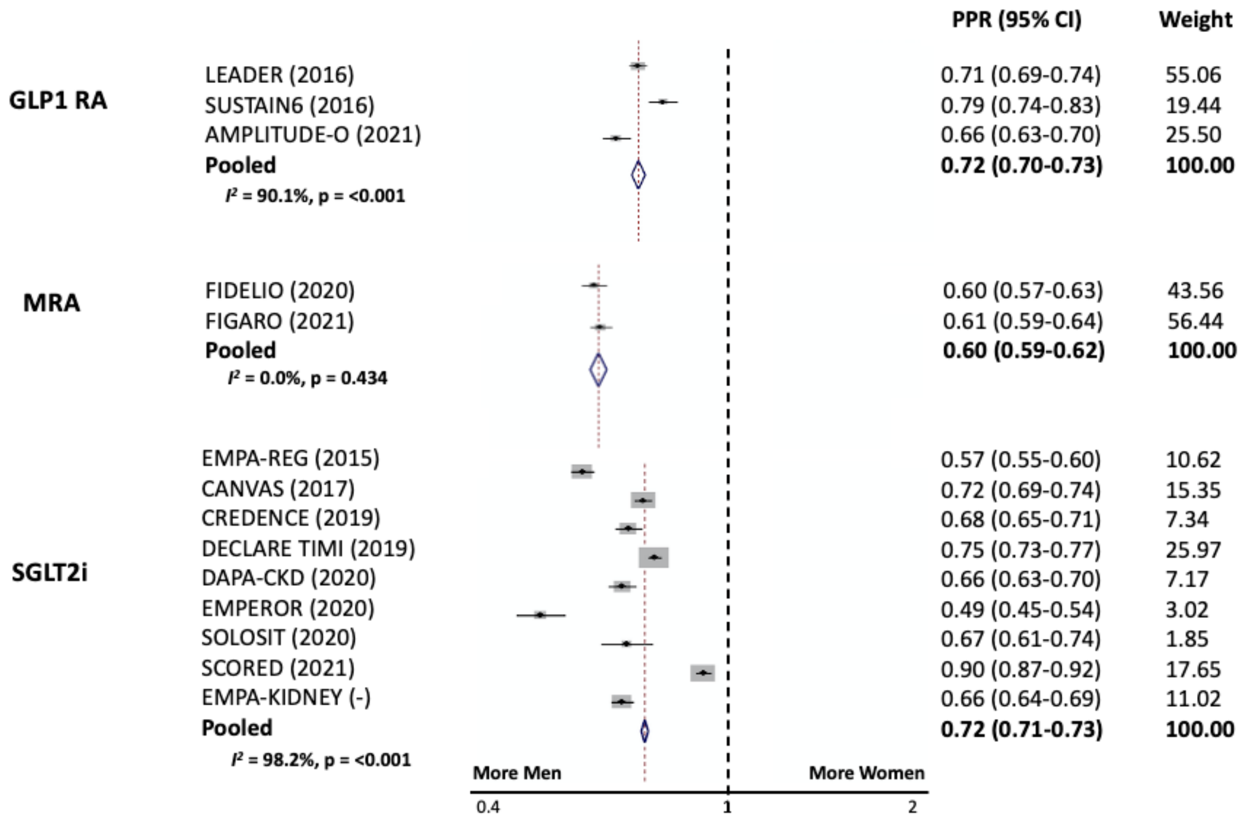

Supplemental Figure 1: Pooled Participation to Prevalence Ratio for Women versus Men in Landmark Trials of GLP1-RAs, MRAs and SGLT-2i from 2015-2021, Assuming Equal Disease Prevalence in Women and Men

Supplemental References

S1. Yakerson A. Women in clinical trials: a review of policy development and health equity in the Canadian context. Int J Equity Health 2019; 18(1): 56.

S2. Jin X, Chandramouli C, Allocco B, Gong E, Lam CSP, Yan LL. Women's Participation in Cardiovascular Clinical Trials From 2010 to 2017. Circulation 2020; 141(7): 540-8.

S3. Scott PE, Unger EF, Jenkins MR, et al. Participation of Women in Clinical Trials Supporting FDA Approval of Cardiovascular Drugs. J Am Coll Cardiol 2018; 71(18): 1960-9.

S4. Steinberg JR, Turner BE, Weeks BT, et al. Analysis of Female Enrollment and Participant Sex by Burden of Disease in US Clinical Trials Between 2000 and 2020. JAMA Netw Open 2021; 4(6): e2113749.

S5. Klein SL. Sex influences immune responses to viruses, and efficacy of prophylaxis and treatments for viral diseases. Bioessays 2012; 34(12): 1050-9.

S6. Soldin OP, Mattison DR. Sex differences in pharmacokinetics and pharmacodynamics. Clin Pharmacokinet 2009; 48(3): 143-57.

S7. Whitley H, Lindsey W. Sex-based differences in drug activity. Am Fam Physician 2009; 80(11): 1254-8.

S8. Solomon SD, McMurray JJV, Anand IS, et al. Angiotensin-Neprilysin Inhibition in Heart Failure with Preserved Ejection Fraction. N Engl J Med 2019; 381(17): 1609-20.

S8. Bots SH, Groepenhoff F, Eikendal ALM, et al. Adverse Drug Reactions to Guideline-Recommended Heart Failure Drugs in Women: A Systematic Review of the Literature. JACC Heart Fail 2019; 7(3): 258-66.

S10. Ladwig KH, Marten-Mittag B, Formanek B, Dammann G. Gender differences of symptom reporting and medical health care utilization in the German population. Eur J Epidemiol 2000; 16(6): 511-8.

S11. Kroenke K, Spitzer RL. Gender differences in the reporting of physical and somatoform symptoms. Psychosom Med 1998; 60(2): 150-5.

S12. Heidari S, Babor TF, De Castro P, Tort S, Curno M. Sex and Gender Equity in Research: rationale for the SAGER guidelines and recommended use. Res Integr Peer Rev 2016; 1: 2.

S13. Peterson ED, Lytle BL, Biswas MS, Coombs L. Willingness to participate in cardiac trials. Am J Geriatr Cardiol 2004; 13(1): 11-5.

S14. Ding EL, Powe NR, Manson JE, Sherber NS, Braunstein JB. Sex differences in perceived risks, distrust, and willingness to participate in clinical trials: a randomized study of cardiovascular prevention trials. Arch Intern Med 2007; 167(9): 905-12.
